# Concurrent Pulmonary Artery Aneurysm, Pulmonary Thrombosis, and Intracardiac Thrombus in Behçet's Disease: A Case Report

**DOI:** 10.7759/cureus.43993

**Published:** 2023-08-23

**Authors:** Othman A Alagha, Ayham Y Abu ElQomboz, Saber F Alsarafandi

**Affiliations:** 1 General Practice, Islamic University of Gaza, Gaza, PSE; 2 Internal Medicine, Alshifa Medical Complex, Gaza, PSE; 3 Pulmonology, Nasser Medical Complex, Gaza, PSE

**Keywords:** intracardiac thrombus, cerebral venous thrombosis, vasculitides, sagittal sinus thrombosis, pulmonary thrombosis, pulmonary artery aneurysm, behcet’s disease

## Abstract

Behçet's disease is a systematic, inflammatory disorder affecting vessels of all sizes. It affects both venous and arterial systems. Vascular involvement carries a high risk of morbidity and mortality. Knowing that Behçet's disease is the most common vasculitides that causes pulmonary artery aneurysms, with a mortality rate of around 25%, makes early detection crucial. Thrombosis in Behçet's disease is mainly caused by an inflammatory process rather than a thrombophilic state, thus vascular thrombosis control is achieved with immunosuppressant medications rather than anticoagulants. An exception to the use of anticoagulants in Behçet's disease appears to be due to cerebral venous thrombosis. The occurrence of multiple site thrombosis and aneurysm simultaneously makes the management very challenging, as we will highlight in our case.

We present a case of a 31-year-old female patient with many prior hospitalizations due to cerebral venous thrombosis, bilateral pulmonary thrombi, right ventricular thrombus, and right pulmonary artery aneurysm. The patient was diagnosed with Behçet's disease according to the Behçet's Syndrome International Study Group criteria and then managed with the prophylactic low molecular weight heparin, cyclophosphamide, and prednisolone, resulting in significant improvement in the patient's symptoms.

Presentation with cerebral venous thrombosis, pulmonary thrombosis, and aneurysm simultaneously is very rare in Behçet's disease. This made this case distinct and challenging in achieving good control of thrombosis and aneurysm simultaneously, which needs close monitoring and a multidisciplinary team to deal with the case.

## Introduction

Behçet's disease is a multi-systematic, inflammatory disorder affecting vessels of all sizes (small, medium, and large) and is classified as systemic vasculitides [1]. This disease is prevalent among young adults aged 20 to 40 years in the Mediterranean, Middle Eastern, and Far Eastern populations. Although there is no difference in prevalence between males and females in the areas where it is more common, females are affected more commonly where it is less prevalent [2-5].

The hallmark manifestation of Behçet's disease is recurrent painful oral ulcers. Other systemic manifestations, including genital ulcers, ocular disease, mucocutaneous involvement, neurologic disease, or vascular disease, can occur [1]. Behçet's disease is unique for its ability to involve blood vessels of all sizes; moreover, it affects both venous and arterial systems. Vascular involvement carries a high risk for morbidity and mortality. More specifically, pulmonary artery aneurysm (PAA) carries a high mortality of nearly 25%, so early recognition is important [6]. Behçet's disease is the most common vasculitides that causes PAAs. Patients with Behçet's disease develop PAA at a younger age compared with patients who have PAA due to another etiology. It is commonly presented with hemoptysis. Also, there is a male predominance with a male-to-female ratio of 3:1 [7].

Patients with vasculitis responded well to steroids and other immunosuppressant medications. Anti-tumor necrosis factor agents such as infliximab and adalimumab have been used successfully in cases where patients did not respond to first-line treatment or developed PAA while on immunosuppressant agents [8,9].

We present a case with a long history of prior hospitalization due to different complaints through a period of two years since her first presentation, diagnosed and managed as a case of Behçet's disease.

## Case presentation

A 31-year-old female patient had no previous medical background. The patient’s history dates back to April 2019 when she started to complain of a headache that was constricting in nature, occipital, lasted all day long, awakened her from sleep, severe 8/10, relieved partially by paracetamol, and associated with vomiting and blurred vision without any history of photophobia, skin rash, or abnormal body movement. After evaluation by a neurologist, brain magnetic resonance venography (MRV) was requested and showed a filling defect in the superior sagittal and transverse sinus suggesting deep sinus thrombosis, and she was diagnosed to have cerebral venous thrombosis affecting the sagittal sinus. An ophthalmology exam showed papilledema. At that time, she was started on warfarin with regular monitoring of the international normalized ratio (INR) at the warfarin clinic and neurology clinic. She was also prescribed acetazolamide for six months. An extensive workup for thrombophilia (antiphospholipid, protein C, protein S, and lupus anticoagulant) was conducted, and the results were negative. Also, serological tests were negative for anti-nuclear antibodies (ANA), rheumatoid factor (RF), perinuclear anti-neutrophil cytoplasmic antibodies (P-ANCA), and antineutrophil cytoplasmic antibodies (C-ANCA). She was doing fine on warfarin treatment, and after six months of follow-up, MRV showed maintenance of blood clots on the superior sagittal sinus, and the neurologist decided on extended therapy.

In July 2021, she developed vaginal bleeding (with clots and no abdominal cramps), dizziness in the form of spinning objects around, and blurred vision. Warfarin was stopped upon recommendation from the gynecologist and internist. The patient was anemic, as shown in Table 1, and she received two units of packed red blood cells (PRBC). She was discharged home undiagnosed, and after two weeks, she started to complain of headache and shortness of breath, and follow-up MRV also showed maintenance of blood clots on the superior sagittal sinus for which she was shifted to apixaban 5 mg twice daily. Urine analysis showed seven to eight RBCs, for which she was stated on prednisolone 10 mg once daily as a suspension of vasculitis, ferrogel tablet, and omeprazole 20 mg once daily.

**Table 1:** Full blood count (July 2021)

Full blood count	
Hemoglobin	7.2 g/dl
Mean corpuscular volume	75.8 fl
Mean corpuscular hemoglobin concentration	33 g/dl
RBC	3 * 10^3 cell/mm^3
Hematocrit	35%
WBC	4.4 * 10^3 cell/mm^3
Platelets	301 * 10^3/mm^3

In November 2021, she was admitted to the female medical ward in Nasser Medical Complex complaining of vague abdominal pain, dyspnea, central and right pleuritic chest pain associated with exertional shortness of breath, and unpleasant awareness of heartbeats. She also complained of oral ulcers (recurrent, painful, multiple, more than three times a year, lasting up to two weeks) and genital ulcers (painful, up to four in count, lasting 10 days in duration, not itchy, non-scarring) that appeared after her initial visit in 2019.

On examination, she was conscious, alert, and oriented, and not in pain or distress. She had a blood pressure of 96/75 mmHg without orthostatic changes, respiratory rate of 24 breaths per minute, heart rate of 110 beats per minute, temperature of 37°C, and O2 saturation of 98% on room air. Chest moved symmetrically with respiration, with no deformity, scar, visible pulsation, or dilated veins. She had a centralized trachea, good chest expansion, and resonant to percussion. On auscultation, there was normal vesicular breathing bilaterally and no wheeze, but right lower basal crepitation was noted. The patient had normal S1 and S2 sounds, regular rhythm, and no murmur or added sound.

**Figure 1:**
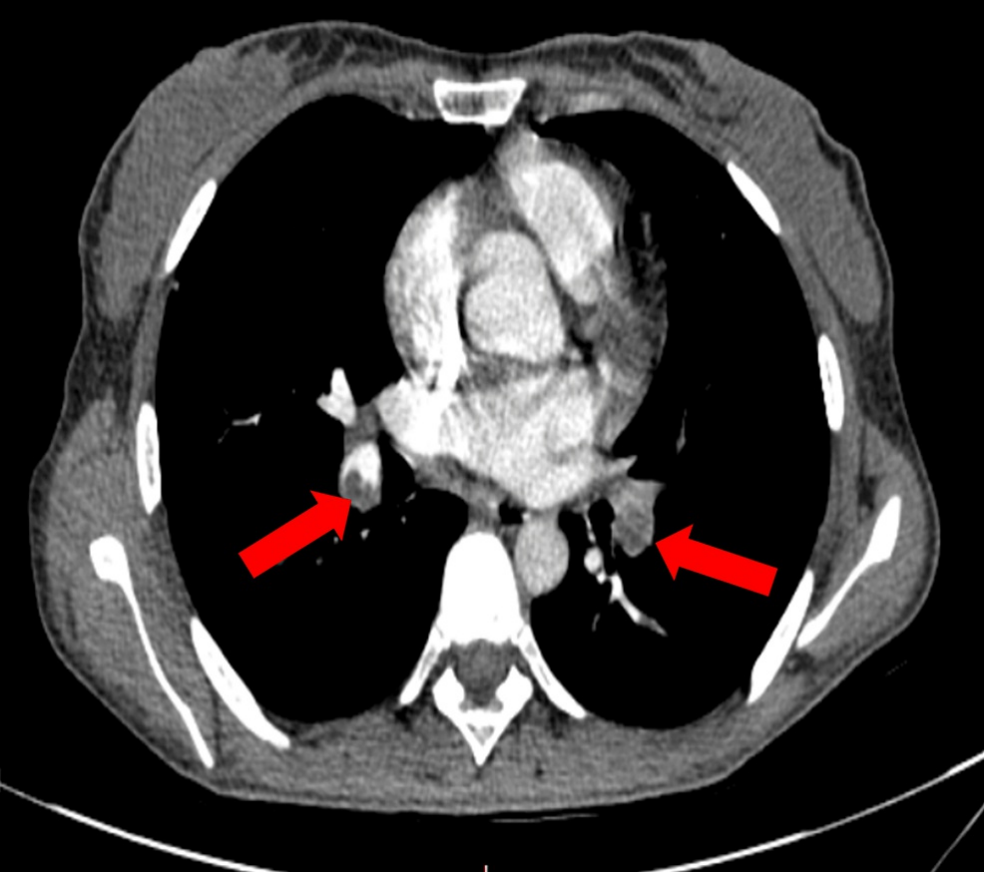
Thorax CT showing bilateral pulmonary artery thrombus with filling defect (red arrows)

**Figure 2:**
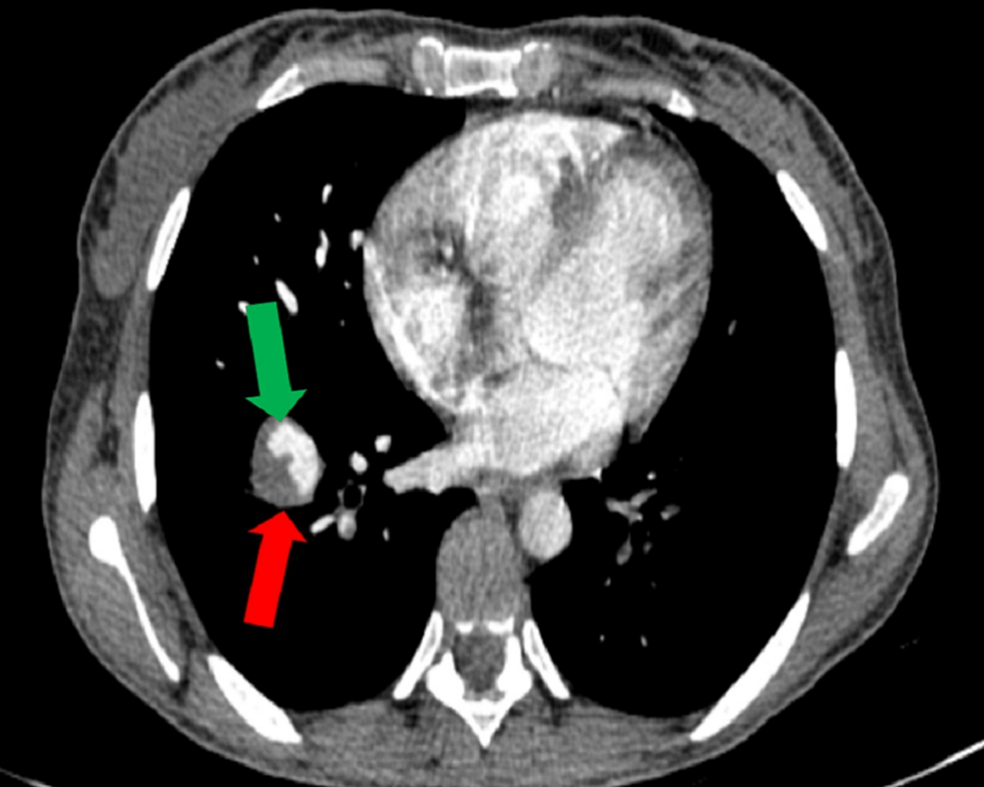
Thorax CT showing pulmonary artery thrombus (green arrow) and aneurysmal dilatation of the right pulmonary artery (red arrow)

A chest CT angiography study was ordered and showed a filling defect in both the right and left segmental and subsegmental pulmonary arteries (Figure 1) and there was a right lower lobe PAA (2.5 x 2 cm) (Figure 2). Echocardiography was done and showed a right ventricular large thrombus (3 x 2.6 cm) and ejection fraction (EF) was 60%, with no valvular abnormality. The left ventricle (LV) was normal in size, with good contraction, and no left ventricular hypertrophy (LVH). The right ventricle (RV) was normal in size and function, with no pericardial effusion.

At this stage, she was diagnosed with Behçet's disease and started on prednisolone 20 mg once daily, warfarin 5 mg once daily, and colchicine 0.5 mg twice daily, and she was discharged home with stable general condition.

**Figure 3:**
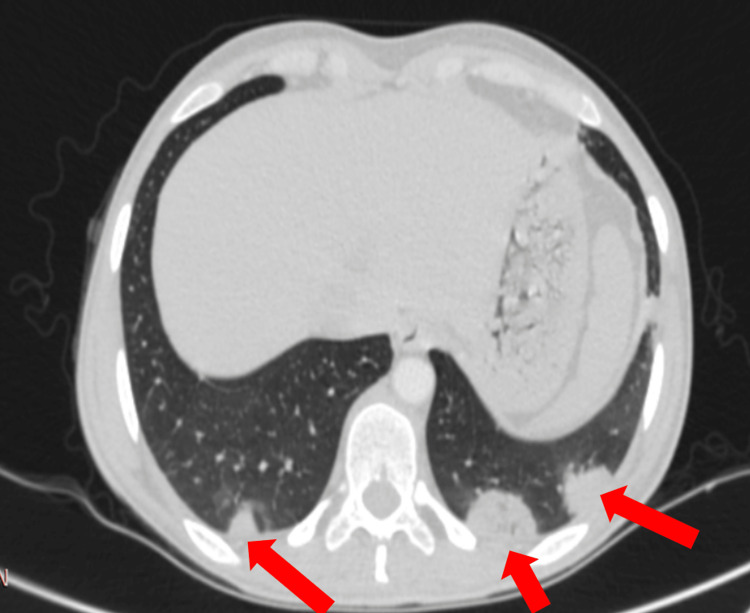
Thorax CT showing consolidated substances related to emboli in the posterior basal segment of the right and left pulmonary lower lobe (red arrows)

In January 2022, she presented to the emergency department with a sudden onset of frank hemoptysis of one-day duration, which was large in amount (roughly 150 ml) and associated with shortness of breath, but there was no palpitation or drowsiness. On examination, she was conscious, alert, and oriented, and not in distress. The patient had a blood pressure of 127/79 mmHg without orthostatic changes, respiratory rate of 22 breaths per minute, heart rate of 107 beats per minute, temperature of 37.5°C, and O2 saturation of 97% on room air. The chest moved symmetrically with a centralized trachea, good chest expansion, and resonant to percussion. On auscultation, there was normal vesicular breathing bilaterally with no wheeze but she had right lower basal crepitation. She had normal S1 and S2 sounds, regular rhythm, and no murmur or added sound. Upon arrival at the hospital, she had no ongoing bleeding, and it resolved spontaneously with minimal hemoglobin drop (nearly 1 g/dl). Follow-up chest CT angiography showed a right-sided segmental branch of PAA increasing in size (3.6 x 3.9 cm), with possible small dissection and multiple bilateral lung infarctions (Figure 3).

We started managing the patient with cyclophosphamide 1 gram on January 5, 2022. The patient was divorced for more than five years and had no recent sexual intercourse according to history. Also, she was started on a pulse steroid for three days (methylprednisolone 1 gram), then switched to prednisolone 60 mg once daily with a tapering dose of 5 mg every two weeks. In view of PAA, embolism, and intracardiac cardiac thrombus, the patient was started on low molecular weight heparin (LMWH) 40 mg subcutaneously once daily. Until recent times, the patient received four doses of monthly cyclophosphamide.

She also underwent video-assisted thoracoscopic surgery (VATS) segmental right pulmonary artery ligation on January 9, 2022, and had a right-side chest drain with no air leak, with a drain level of 70 cc serous.

During the follow-up, the patient received four doses of monthly cyclophosphamide and a tapering dose of prednisolone. There was a significant improvement in the patient's symptoms in the form of right pleuritic chest pain, dyspnea, and palpitation, and there was no headache or blurring of vision. Follow-up echocardiography showed significant improvement in the intracardiac thrombus size, and there were no newly formed emboli. Also, pulmonary infarction regressed. This situation showed the effectiveness of the treatment regimen. The patient was maintained on LMWH, cyclophosphamide, and prednisolone.

## Discussion

We described a case of a 31-year-old female patient with recurrent hospitalizations due to cerebral venous thrombosis, bilateral pulmonary thrombi, right ventricular thrombus, and right PAA. The patient was diagnosed with Behçet's disease and managed with prophylactic LMWH, cyclophosphamide, and prednisolone, resulting in significant improvement in the patient's symptoms.

Presentation with cerebral venous thrombosis, pulmonary thrombosis, and aneurysm simultaneously is very rare in Behçet's disease, making this case distinct and challenging in achieving good control of thrombosis and aneurysm simultaneously. Our case presented another patient who had multiple site thrombi and PAA, who was successfully managed with steroids and immunosuppressants in addition to many cases in the literature that we will discuss below.

As mentioned previously, Behçet's disease is a systemic vasculitis manifested with different systemic symptoms and presentations. Vascular involvement is serious and carries a high risk for morbidity and mortality. More specifically, PAA carries a high mortality of nearly 25%, so early recognition is important [6]. Although men are more likely to have vascular manifestations, women are more likely to have arterial disease. The types of vascular involvement are different, including superficial thrombosis, deep vein thrombosis, and aneurysm formation on the arterial side thrombosis, considering the first is the most common while the latter is the rarest, respectively. Patients with vascular manifestations of Behçet's disease are more likely to have cardiac involvement [6,10].

PAA is a very rare entity caused by various etiologies, such as infections, cardiac and vascular anomalies, and Behçet's disease, which is the most common vasculitis that causes PAA [11,12]. The incidence of patients with Behçet's disease who develop PAA is not clearly defined. In a single-center study involving 2179 patients, 1.1% of patients were diagnosed with PAA [13,14].

Patients with vasculitis responded well to steroids and other immunosuppressant medications. Anti-tumor necrosis factor agents, such as infliximab and adalimumab, have been used successfully in cases where patients did not respond to first-line treatment or developed PAA while on immunosuppressant agents [8,9]. Immunosuppressant treatment alone has been successful in the treatment of large aneurysm [15]. In a case of Behçet's disease with a large aneurysm, complete thrombosis resolution was achieved within 18 days of starting pulse steroids and cyclophosphamide [16]. In people with Behçet's disease, right ventricular thrombus can also be simultaneously present in rare cases requiring extended periods of anticoagulation in addition to immunosuppressants [17].

In general, control of vascular thrombosis is achieved with immunosuppressant medications rather than anticoagulants. Thrombosis in Behçet's disease is mainly caused by an inflammatory process rather than a thrombophilic state, making anticoagulants not effective in preventing recurrent thrombotic events. An exception to the use of anticoagulants in Behçet's disease appears to be due to cerebral venous thrombosis [11]. However, in selected patients for whom anticoagulation seems a useful treatment to add to immunosuppressants, it is strongly recommended to perform a lung CT scan before starting such therapy, to rule out the presence of occult PAA and to prevent the consequent risk of rupture and bleeding [18].

In our case, the patient's condition was discussed with a multidisciplinary team (pulmonologist, internist, and cardiologist) giving a history of recurrent vaginal bleeding and a possible increase in the PAA size while the patient was receiving warfarin as a treatment for pulmonary embolism and intracardiac thrombus. Warfarin was not the preferred treatment option due to the risk of bleeding and the possible cause of increasing aneurysm size.

The use of anticoagulants in case of cerebral venous thrombosis due to Behçet's disease has shown to be effective. Therefore, the patient was started on LMWH 40 mg once daily as a prophylactic dose. Also, the patient received monthly cyclophosphamide and prednisolone 60 mg once daily. As thrombosis in Behçet's disease is mainly caused by an inflammatory process rather than a thrombophilic state, control of vascular thrombosis is achieved with immunosuppressant medications rather than anticoagulants.

Anti-tumor necrosis factor agents, such as infliximab and adalimumab, have been used successfully in cases with PAA; unfortunately, we do not have such medications in Gaza due to limited resources and we gave the patient a referral to the West Bank to receive infliximab but she cannot reach there.

The patient underwent VATS segmental right pulmonary artery ligation later on after referral to West Bank.

## Conclusions

Behçet's disease is a multi-system vasculitis affecting all vessel types. The first presentation for patients with Behçet's disease varies widely according to the affected systems and vessels. Although presentation with pulmonary thrombosis and aneurysm simultaneously is very rare in Behçet's disease, it has a high morbidity and mortality rate and should be detected as early as possible. Thus, taking the history of patients thoroughly and a high index of suspicion are needed for physicians working in areas that have a higher incidence of Behçet's disease. Steroids and immunosuppressants showed a good result in managing thrombosis due to Behçet's disease.
